# Progress of the Medical Sciences

**Published:** 1901-12

**Authors:** 


### Progress of the Medical Sciences.

### MEDICINE.

**Formation of Red Hæmoglobin and of Corpuscles of the Blood.**—Aporti[1] found that by feeding healthy dogs on an iron-free diet, and by bleeding them freely, a proportionate fall in the amount of hæmoglobin and of red blood corpuscles took place. After a certain number of bleedings the hæmoglobin remained at this lower level or still further decreased, whilst the red corpuscles gradually increased again. Injections of arsenic into these animals caused an appreciable increase in the number of the red corpuscles, whilst the amount of hæmoglobin remained unaltered. On the other hand, injections of iron did not affect the number of red corpuscles, but caused a rapid increase in the quantity of hæmoglobin. According to Aporti, there is therefore a certain amount of independence in the formation of red corpuscles and of hæmoglobin, and some substances, especially arsenic, increase the number of red cells; whilst others, iron *par excellence*, almost exclusively affect the formation of hæmoglobin.

[1] *Centralbl. f. innere Med.*, 1900, XXI. 41.

**The Rôle of Iron in Blood Formation.**—Hofmann,[1] in observations on the various new preparations of iron, showed that they were as well, but not better, absorbed than the ordinary inorganic salts of iron. The particular preparation does not matter, provided that it contains a sufficient quantity of iron. The use of preparations of hæmoglobin is so far wrong that the quantity of iron present in them is small, and very large doses must be taken to reach the amount contained in small doses of other preparations. He looks upon chlorosis as due to functional incapacity of the blood-forming organs of the bone marrow, either appearing at puberty or congenital, which in severe cases is combined with hypoplasia of the vessels (Virchow) and of the sexual apparatus. This weakness of the blood-forming organs is expressed in the lessened amount of hæmoglobin and altered (diseased) form of the red corpuscles.

**Red Blood-Corpuscles and Hæmoglobin in the Pregnant and New-born.**[2]—At the end of pregnancy in the mother, the amount of hæmoglobin is lessened by an average of 20 per cent., number of red blood cells by about 500,000 per c.mm. In women at the 7½ to 8½ month of pregnancy the numbers are rather higher. In new-born infants, on the average, the hæmoglobin is 120 per cent., and the red blood cells 6½ millions per c.m. In prematurely-born infants the numbers are still higher. In the blood of new-born infants, especially of those born prematurely, there are numerous red blood corpuscles (microcytes) which stain with methyl-blue, and, according to the authors, are probably young forms.

**Leucocytes in Pregnancy.**—As the result of numerous observations, Nannicini[3] finds that in multiparæ there is practically no leucocytosis during pregnancy; although the white corpuscles are somewhat increased, they are within physiological limits. In primiparæ the number of the white cells is so large that it is justifiable to speak of a "Leucocytosis Gravidarum." The number of white corpuscles bears no relation to the period of pregnancy. In neither primiparæ or multiparæ is there a digestive leucocytosis; the number of leucocytes in the blood may even be less during the period of digestion.

**Leucopenia is present in cases of influenza, when these are of the typhoid type,** just as is commonly observed in enteric fever apart from complications such as pneumonia. So in cases of tuberculosis affecting the abdominal organs there is no leucocytosis, and often there is leucopenia. Many authors consider that the leucocytosis of infectious diseases is due to the passing out of the leucocytes from the lymph-glands from chemiotactic attraction. The chief lymphatic organs below the diaphragm being the spleen, the mesenteric and post-peritoneal glands, the simple follicles and Peyer's patches in the intestine, it is sugges-

[1] *Arch. f. path. Anat.* [etc.], 1900, clx. 235.
[2] Bidone and Gardini, *Arch. ital. de Biol.*, 1899, xxxii. 36.
[3] *Settimana med.*, 1890, liii. 32, 33.

tive that in those infectious diseases in which the functions of these glands are disturbed (enteric fever, intestinal tuberculosis, influenza of typhoid type) leucocytosis does not occur, just as in extensive affection of the intestinal glands no digestive leucocytosis takes place.[1]

**Blood-regeneration in Chlorosis.**—Schauman and v. Willebrand[2] found that under the treatment employed by them the improvement in the blood-state was shown rather in a rapid increase of the number of corpuscles than in their staining property. In the course of convalescence the average diameter of the red corpusles increased until it reached a maximum; this increase was due to a numerical decline of small as well as to an increase of large cells. In contrast to pernicious anæmia, in which during the first half of convalescence the red corpuscles stain as deeply or more deeply than normal, in chlorosis and traumatic anæmia the newly-formed erythrocytes are as a rule more or less deficient in staining material.

**The Clinical Study of Anæmias with Leucopenia,** by Decastello and Hofbauer.[3]—In spite of the difference in the etiology of the anæmias, there was in all cases, in which there was no disease of the glands, an increase in the percentage of lymphocytes present, and a corresponding decrease in the number of the neutrophil polymorphonuclear leucocytes. Leucopenic anæmias, which were complicated with diseases of the glands (spleen, lymph-glands), showed, in relation to the percentage of polymorphonuclear and mononuclear elements, a varying condition. The authors consider that leucopenia, as a sequel of anæmic states, depends upon injury to the leucocyte-forming organs, especially to the bone-marrow. Leucopenia is not, however, necessarily a sign of very bad omen, for it is found in some less serious diseases.

**In 110 cases of pernicious anæmia** analysed by Cabot[4] there were 57 men and 53 women, of whom 28 were under 40 years of age. No certain influence in causing the disease could be attributed to occupation, the menopause, syphilis, or intestinal parasites. Hemorrhages were present in 37 cases. The severity of the symptoms did not correspond with the blood-changes. Patients with 500,000 red corpuscles to c.m. often felt better than those with 1½ millions. On this account and on other grounds the author is of opinion that the disease is due to the action of a poison invading the body from the intestinal tract. In all cases under 2,000,000 red corpuscles to c.m. were found: in most there was a relatively high amount of hæmoglobin, in contradistinction to equally severe cases of secondary anæmia. Secondary and pernicious anæmia are two distinct diseases. The anæmia produced by the presence of the bothriocephalus cannot, however, be distinguished from per-

[1] Blum, *Wien. klin. Wchnschr.*, 1899, xii. 15.
[2] *Berl. klin. Wchnschr.*, 1899, xxxvi. 1, 3.
[3] *Ztschr. f. klin. Med.*, 1900, xxxix. 488. [4] *Am. J. M. Sc.*, 1900, cxx. 139.

nicious anæmia. The duration of the disease in most of the cases was less than two years. There are, however, cases of pernicious anæmia which run a mild course, and last even for six years, with temporary and striking ameliorations, independent of treatment.

**The Metabolism in Severe Anæmia.**—In four cases von Moraczewski[1] investigated the metabolism with reference to P, Cl, and Ca. A typical change was the small power of assimilation of the body, as evidenced by the slight nitrogen loss on a diet relatively poor in nitrogen, and still more clearly from the nitrogen-retention if N is added to the diet. This N retention is pathological, it is not due to good nutrition, but to bad dis-assimilation. The Cl retention is not so striking as in anæmias due to carcinoma and in chlorosis. In all cases there was an absolute loss of Ca. The excretion of Ca (and of Cl) is to be considered in relation to the fact that in pernicious anæmia the bones, and especially the medulla of the bones, are altered. Treatment with calcium salts had a good effect. In diagnosis it is probable that a case is one of pernicious and not of another form of anæmia, if there is a relatively large Cl excretion and an absolute Ca loss together with a lessened N and P excretion.

**The Blood at High Altitudes.**—Most authors who have studied the question of the condition of the blood during residence at high altitudes have found an increase of the red blood cells, as well as an increased amount of hæmoglobin, as a result of such residence. Jaquet, whose former researches had led him to the conclusion that the volume of the blood and quantity of hæmoglobin was increased, has lately[2] endeavoured to distinguish which of the chief factors present at mountain resorts—temperature, humidity, and air-pressure—is responsible for the blood-changes. The experiment was carried out on growing puppies of the same age, and the total amount of hæmoglobin and blood was estimated by washing out the body (blood-vessels) at the end of the experiment. 1. Temperature: No difference was found in the blood of animals kept at 2°–5°C., and those kept at 13°–16°C. under the same air-pressure and humidity for a period of six weeks. 2. Air-pressure: By means of special arrangements, three puppies were kept for four weeks under a pressure of 640 mm. Hg; a lowering of pressure of 100 mm. Hg, corresponding to the height of Davos. At the conclusion of the experiment a considerable increase was found both in the number of blood corpuscles (more than 1.5 millions) and also in the percentage of hæmoglobin (248 per cent.). On the effect of increased or decreased humidity Jaquet has not experimented, but from a critical study of the literature he concludes that the influence on blood-formation cannot be pronounced.

[1] *Arch. f. path. Anat.* [etc.], 1900, clix. 221.
[2] *Arch. f. exper. Path. u. Pharmakol.*, 1900, xlv. 1. *u.* 2.

**New Test for Blood in Urine.**—To 10 cc. urine add 1 cc. ammonium sulphide and as much pyridin. According to the amount of blood present the fluid takes a more or less orange-red colour. With considerable quantities of blood this colour-change is constant. Although this colour-reaction is very sensitive, it is considerably surpassed in delicacy by the observation by means of the spectroscope of the hæmochromogen formed. The spectrum of hæmochromogen is quite distinct, even if no naked-eye colour-reaction can be distinguished.[1]

**For the presence of blood in urine** the well-known test of a blue colour with tinct. guaiaci and ozonised turpentine is often employed. Pus in urine gives a blue colour with tinct. guaiaci without the addition of any oxygen-giving body. As the urine may contain reducing bodies, which make the appearance of the blue colour difficult, it is best to filter the urine and do the test on the filter. In like manner in leukæmia drops of blood dissolved in water and filtered give a blue reaction on the filter with tinct. guaiaci. This property of pus of giving a blue colour with tinct. guaiaci depends on the action of nucleo-proteids, and is in all probability to be attributed essentially to the leucocytes and not to the lymphocytes.

NOTE.—For the above abstracts I am indebted to an article by Dr. R. Forstmann, "Neuere Arbeiten aus dem Gebiete der Physiologie und Pathologie des Blutes."—*Schmidt's Jahrb.*, 1901, cclxx. 138, 225.

J. MICHELL CLARKE.

### SURGERY.

The **surgery of the pancreas** is now coming into line with that of the rest of the abdominal viscera, though the anatomical relations of the organ and its deep situation in the abdomen must necessarily prevent such radical treatment in the removal of malignant tumours as can be elsewhere adopted.

In the treatment of **pancreatitis** the greatest advance has been made, though the diagnosis and treatment of the acute forms presents much difficulty at present. The chronic forms, though frequently mistaken for malignant disease of the head of the pancreas or for gallstones in the common duct, have yielded a success beyond one's expectation. To open the abdomen in cases of advanced cholæmia and jaundice with enlargement of the head of the pancreas, and then to find that a cure is effected by draining the gall-bladder, is calculated to set one thinking as to the cause of the pancreatic enlargement. Such unexpected results have been largely instrumental in laying the foundation of the treatment of chronic pancreatitis. An address on pancreatitis,[2] delivered before the American

[1] Donogány, *Arch. f. path. Anat.* [etc.], 1897, cxlviii. 234.

[2] *Brit. M. J.*, 1901, i. 1129.

Surgical Association by Mr. Mayo Robson sets before us the chief points in the diagnosis and treatment of this affection.

That the pancreatic duct should be subject to an infective or suppurative inflammation is not surprising, when we remember that it opens with the common bile duct into the second part the duodenum, which usually contains septic organisms, especially when any catarrhal duodenitis is present. An obstruction of the common bile duct is shown by jaundice, but we have no pathognomonic sign of an occlusion of the pancreatic ducts, though extremely rapid loss of weight and fat necrosis would afford some clue. By fat necrosis is understood a splitting up of the fat into fatty acids and glycerine: the latter is absorbed; but the acids, being insoluble, remain in the cells and unite with calcium salts, forming yellowish-white patches of various sizes in the subperitoneal fat and in the omentum, mesentery, &c.

Hitherto, however, fat necrosis has only been discovered after the abdomen has been opened; but Robson thinks that physiological chemistry may perhaps help us in the diagnosis of these difficult cases by an examination of the urine alone, or of the urine, the blood, and the fæces. Glycosuria, lipuria, and fat in the stools are helpful in the diagnosis when they are present, and in one case I was able to make a correct diagnosis from the presence of sugar in the urine, but they occur too seldom to be of great practical value.

It is well known that there is a peculiar relation between pancreatic disease and serious hemorrhage, and the subject of hemorrhagic pancreatitis has received attention in this *Journal*[1] and elsewhere. Robson, after carefully considering the whole subject, has come to the following conclusions as regards hemorrhage in diseases of the pancreas:—1. That in certain diseases of the pancreas there is a *general* hemorrhagic tendency, which is much intensified by the presence of jaundice. 2. That hemorrhage may apparently occur in the pancreas unassociated with inflammation or with jaundice, or with a general hemorrhagic tendency. 3. That both acute and chronic pancreatitis can and do frequently occur without hemorrhage. 4. That some cases of pancreatitis are associated with local hemorrhage. The injection of glycerine is sometimes followed by hæmaturia, and Robson suggests that the glycerine set free in the tissues by the fat necrosis may possibly be the real cause of the local hemorrhagic tendency in pancreatic affections. In discussing these matters with Mr. Cammidge, it was suggested that the glycerine set free would become oxidised in the blood just like any other alcohol, and the oxidation product expected would be an aldehyde, but the tests employed failed to demonstrate this. In the course of these investigations, however, Mr. Cammidge found that if the urine was boiled for a short time with an oxidising agent, and then the phenyl-hydrazin test performed, an abundant crop of delicate yellow needles arranged

[1] 1897, xv. 44.

in sheaves and rosettes was produced. The untreated urine gave no such result; normal urine, morbid urines from gout, &c., and, most important, bilious urine from patients suffering from simple catarrhal jaundice, gave also negative results. The application of this test gave confirmatory results in two of Robson's cases—one of chronic pancreatitis and one of stone in the common duct without manifest affection of the pancreas—the crystals being obtained in the former, but not in the latter case.

**Acute pancreatitis** is accompanied by pain and vomiting, and simulates intestinal obstruction, leading to efforts to secure an evacuation of the bowels and relief to the distension, as in a case I operated upon at the Royal Infirmary a few months since. The treatment practically resolves itself into that of peritonitis, commencing in the epigastrium; but death nearly always occurs in cases in which the diagnosis of acute pancreatitis is confirmed.[1] (Richardson.)

An exploration should be undertaken through the mid-line in front above the umbilicus. After establishing the diagnosis by this means, a posterior incision, which must be a free vertical one in the left costo-vertebral angle so as to permit the insertion of the whole hand if necessary, will enable the diseased organ to be freely examined, and if necessary drained for the evacuation of pus and gangrenous material, without involving risk to the general peritoneal cavity. Robson thinks such an operation as imperative as the early evacuation of septic matter in perforative or gangrenous appendicitis.

**Subacute pancreatitis** is fortunately more amenable to treatment, as the symptoms are not so urgent as in the acute form. As a rule abscess formation occurs before operation is undertaken, but this is by no means necessary. If surgical treatment is decided upon, a median incision may be made in the middle line for exploratory purposes, and drainage effected through the costo-vertebral angle as described above. If the posterior incision is regarded as impracticable, the abscess may be aspirated and the cavity opened and packed with gauze, which may be brought forward through a large rubber tube, which will become isolated from the general peritoneal cavity in the course of forty-eight hours.

In **chronic pancreatitis** the drainage is indirect, and obtained by draining the gall-bladder by cholecystotomy, cholecystenterostomy, or duodeno-choledochotomy. Cholecystotomy has been performed in most of Robson's successful cases. Gallstones, if present, should be removed, and the drainage must not be stopped before the bile has become healthy and not before the greater amount of bile is being passed by the bowel. Experience seems to show that cholecystenterostomy is not an ideal operation in the treatment of these cases. Barling[2] records

[1] *Boston M. & S. J.*, 1901, cxliv. 235; abstract in *Med. Rec.*, 1901, lix. 425.

[2] *Brit. M. J.*, 1900, ii. 1766.

a case in which the gall-bladder was joined to the duodenum with resulting cure of the jaundice and the other symptoms complained of, but the pancreas still remained enlarged. It is desirable to emphasise Robson's statement that the simulation of malignant disease of the head of the pancreas by chronic interstitial pancreatitis should not deter us from declining to operate in any case of distended gall-bladder when the patient is in a condition to bear it, or even in any case of chronic jaundice without distention of the gall-bladder where the general health is deteriorating, as, though it should be recognised that if the disease be really malignant very little good will be done and life may even be shortened or only prolonged for a short time, yet if the disease prove to be chronic pancreatitis a real and permanent cure may be brought about. If a calculus be felt embedded in the head of the pancreas or impacted in the pancreatic duct, it may be reached through the second part of the duodenum by laying open the papilla and exploring the duct, or by dividing the peritoneum passing between the duodenum and hepatic flexure of the colon and then cutting through the overlying pancreas on to the concretions. If the papilla common to the bile and pancreatic ducts be incised in the duodenum it does not require suture, and in the cases in which Robson has explored the ducts by the duodenal route there has been no serious hemorrhage; the anterior duodenal opening only requires closing by a mucous and a serous suture. Drainage of the right kidney pouch for twenty-four to forty-eight hours is generally advisable. Both in Robson's[1] and Barling's[2] cases the results have been most encouraging, and only one patient died as the result of the operation. The treatment of pancreatitis may, therefore, be regarded as established on a firm basis, and further developments may be expected.

James Swain.

### LARYNGOLOGY AND RHINOLOGY.

**Nasal Respiration.**—Nasal obstruction is often complained of by patients in whom examination fails to reveal any obvious cause, while, on the other hand, when partial stenosis of the passages does exist it frequently passes almost unnoticed by the patient; in other words, there is little correspondence between the degree of nasal stenosis and the subjective sense of obstruction to nasal respiration. Everyone has experienced the disagreeable sense of stuffiness in the nose which accompanies a cold in the head, which persists however freely the nose is "cleared" by blowing, and when actual stenosis is certainly non-existent. Buccal respiration in patients with adenoid growths, despite the prevalent belief to the contrary, is similarly due to the associated rhinitis rather than to actual occlusion of

[1] *Loc. cit.* [2] *Loc. cit.*

the nasal passages by the adenoid growths. Explanation of this clinical paradox is afforded by C. A. Parker's [1] observations on the air-currents in nasal respiration, by making patients breath naturally through the nose air impregnated with lycopodium powder. By noting the parts where the powder was most thickly deposited, the path of the inspired air through the nose was readily determined. These experiments proved that in normal noses the air impinges first on the anterior part of the nasal septal cartilage and then shoots upwards and backwards to the olfactory fissure, thence passing between the middle turbinated body and superior meatus and the corresponding portion of the septum; that is to say, the backward current of inspired air is along the highest part of the nasal passages. Very little air passes along the level of the middle meatus, and practically none along the inferior meatus. Posteriorly the powder was mainly deposited quite at the top of the choanæ, the upper portion of the septum, on the roof of the naso-pharynx, and on the Eustachian cushions. Expiration was determined by observing smoke puffed out through the nose, and appeared to be largely confined to the lower meatus. Hence very slight reductions of the anterior ends of the middle or inferior turbinate bodies, the removal of small polypi in the middle or superior meatus, or the cure of a chronic nasal catarrh, will often do far more to relieve a patient than more radical measures, which are too often addressed to the inferior turbinates, or than the removal of slight septal spurs or deviations which are outside the normal air paths.

**Tuberculosis of the Larynx.**—Too much and too little are claimed for therapeutical methods in laryngeal tuberculosis. The disease can only be eradicated in a relatively few favourable cases; but for the latter even lasting cures may often be obtained, and in a larger proportion the disease can be arrested for a time at any rate.

In opening a recent discussion on the local treatment of this affection, Middlemass Hunt admitted that all the bright hopes with which the writings of Krause and Heryng inspired us have not been realised, nevertheless "it is no exaggeration to say that thousands of lives have been prolonged and an incalulable amount of suffering averted as a result of the work which has been done in this field." [2] Palliative treatment, consisting of antiseptic and local anæsthetic applications, are alone desirable whenever there is extensive ulceration or infiltration, especially with much œdema or perichondritis, particularly if associated with high fever, loss of appetite, and advancing lung disease. But when ulceration is present, is superficial and not too extensive, in cases otherwise suitable, the application of strong lactic acid, varying in strength from 50 per cent. to the undiluted acid, should be well rubbed into the ulcerated surface once a week or a fortnight; if this strength of acid cannot be

[1] *J. of Laryngol.*, 1901, xvi., 345. [2] *Brit. M. J.*, 1901, ii., 877.

tolerated, it may be employed in weaker solutions of 20 to 30 per cent. Deep granulating ulcers of limited extent heal more rapidly when thorough curettement is done before the acid is applied. Barclay Baron[1] urged the use of inhalations of benzoin, creasote, menthol, &c., or intratracheal injections of guaiacol and menthol, but deprecated resorting to submucous injections. Lake[2] has obtained good results in some cases of localised deposit by excision with cutting forceps prior to applications of lactic acid. Watson Williams[3] emphasised the positive value of submucous injections of guaiacol in almond oil, biniodide of mercury in aqueous solution, and other germicides, where there was no ulceration, and where therefore it was undesirable to create an ulcerating surface. By such measures he had obtained lasting arrest of the laryngeal disease in several cases, although curettement and the local application of lactic acid of full strength still holds the first place in the radical treatment of laryngeal tuberculosis in the practice of the great majority of laryngologists.

Orthoform is proving a great rival to cocaine as a local anæsthetic in this disease. Sendziak[4] finds that it is really an excellent drug, which, applied either in the form of powder or in connection with menthol (Freudenthal: Menthol, 1.0 to 5.0, 10.0 to 15.0; ol. amygd. dulc., 30.0; vitelli ovorum, 25.0; orthoform, 12.0; aq. dest. q.s. ad. 100.0: fiat emulsio), by means of brushings or laryngeal syringes, produces anæsthesia and relief from pain, lasting usually a couple of hours, and at times as long as twenty-four hours. Sendziak has not only noticed an analgesic action in cases treated with orthoform, but also a favourable action upon the tuberculous lesions themselves, so that he regards "this drug as a very precious acquisition in the local therapy of laryngeal tuberculosis." Similar encomiums on the beneficial effect of orthoform were expressed by McCall,[5] especially when the drug was associated with resorcin. This combination, in proportions varying from one-third to two-thirds, applied every second day, had given him excellent results in cases with ulceration and exuberant granulations, such cases as are usually curetted. In flat superficial ulcerations, such as occur in the epiglottis, for instance, orthoform, combined with bismuth, morphine, or cocaine, answers better in his experience.

P. WATSON WILLIAMS.

### OTOLOGY.

The "**complete mastoid operation**" has received a new impetus by the work of Mr. Charles A. Ballance. In an interesting and important paper[6] read before the Royal Medical and Chirurgical

[1] *Ibid.* [2] *Ibid.* [3] *Ibid.*
[4] *J. of Laryngol.*, 1901, xvi., 223. [5] *Brit. M. J.*, 1901, ii. 881.
[6] *Med.-Chir Tr.*, 1900, lxxxiii. 125.

Society on January 23rd, 1900, this surgeon brought forward a modification of the operation, which was received with much favour, and which is being extensively practised at the present time. Mr. Ballance began by pointing out that the successful conduct of the operation for chronic otorrhœa requires the fulfilment of two conditions: (1) The removal of all disease by operation; (2) the healing of the large wound from the bottom.

In order to carry out the second condition, it had been necessary to tampon the cavity at frequent intervals with strips of dry antiseptic gauze. This process, however carefully carried out, is painful; and as it requires to be continued for many weeks or months before cicatrisation is complete, it is often neglected, and the result of the operation is not satisfactory. In order to obviate the painful, prolonged, and unsatisfactory after-treatment of the operation, he proposed that chronic otorrhœa should be treated by two operations instead of one. The first operation he would call *the operation for the removal of the disease;* the second operation he would call *the operation for the healing of the wound.* The latter would be attained by the epithelial grafting of the tegmina antri and tympani, of the inner walls of the antrum, attic and tympanum, and of the fresh-cut bone forming the anterior and superior walls of the inner extremity of the enlarged osseous meatus.

*Description of First Operation.*—The incision is made behind the pinna, and the latter held forward with a rake retractor. A round cross-cut burr, driven by an electric motor, is used to open the bony cavity, but when no motor or burr is available a gouge is recommended; and while this is being used a Stacke's protector serves to shield from injury the tuberosity which projects from the inner wall into the neck of the antrum, and contains the Fallopian aqueduct and the horizontal semicircular canal. The posterior wall of the osseous meatus is removed with a pair of small, angular, bone-cutting forceps. Sharp spoons of various sizes are used to remove the diseased granulations and soft parts from antrum, attic and tympanum. The tegmina antri and tympani, and the inner walls of these cavities, should be left by the spoon clean and hard. A bright light is required to do this well, and temporary plugging with dry gauze is an important aid. The cartilaginous canal is next dealt with. Its inferior wall is divided vertically well into the concha. The cut in the concha is then carried with a curve upwards and backwards till it reaches the level of the anterior commencement of the helix. The posterior wall of the meatus is then pushed upwards and backwards, and attached in a special manner by one, two, or three silkworm-gut threads to the mastoid flap. The bone cavity is then plugged with gauze through the meatus.

Under favourable circumstances the second operation may be done a week later, but in adults not for two or three weeks.

*Description of Second Operation.*—An anæsthetic having been given, the original incision is again opened; this being easily accomplished by the handle of the knife. The pinna is now displaced forwards, and care taken to arrest all oozing from the granulating surface. The tympano-antral cavity must be rendered quite dry by little pieces of gauze held on forceps. Large epithelial grafts, as thin as possible, are now taken from the thigh or arm. If the surgeon is successful in cutting an epithelial graft large enough to cover the whole area of the granulating surface, it can be applied with advantage in one piece. The graft is best carried to the wound spread out on a microscope section lifter. It should be made to cover—(1) The anterior wall of the cavity formed internally by the anterior boundary of the tympanum and attic, and externally by the anterior wall of the enlarged osseous meatus; (2) the anterior part of the roof of the cavity formed by the tegmen tympani and the superior wall of the enlarged osseous meatus; (3) the inner walls of the attic and tympanum; (4) the tegmen antri; (5) the ridge formed by the Fallopian aqueduct; and (6) the inner wall of the antrum.

As an aid in pressing the epithelium graft firmly into the cavity, steel probes or stoppers are recommended with pear-shaped heads. As a protective to the grafts, gold-leaf should be applied over them and carefully pushed into position, after which a narrow strip of dry iodoform gauze is packed into the cavity and allowed to remain for a week. The gold-leaf may be left undisturbed for three or four more days; it should then be removed with forceps. Twenty cases are recorded, all more or less successful.

Dr. William Milligan, of Manchester, read a paper[1] at the section of otology at the recent Association meeting at Cheltenham, bringing forward some improvements in the above operation, recommending especially that, in order to avoid the constant oozing of blood at the second operation, the original incision should be opened the day before the second or grafting operation. He also recommended that the graft or grafts should be floated into position by filling the antro-tympanal cavity with warm salt solution. The graft is floated upon this fluid and the fluid sucked out by a pipette. He also preferred glass stoppers to those made of steel for pressing the grafts into position. Milligan's general experience of the grafting operation has been satisfactory.

W. H. HARSANT.

[1] *Brit. M. J.*, 1901, ii. 893.

